# Management of Inoperable Supra-Sellar Low-Grade Glioma With BRAF Mutation in Young Children

**DOI:** 10.7759/cureus.19400

**Published:** 2021-11-09

**Authors:** Kaitlyn Howden, Stacy Chapman, Demitre Serletis, Colin Kazina, Mubeen F Rafay, Damien Faury, Lili-Naz Hazrati, Nada Jabado, Magimairajan Issai Vanan

**Affiliations:** 1 Section of General Pediatrics, Department of Pediatrics and Child Health, CancerCare Manitoba, University of Manitoba, Winnipeg, CAN; 2 Section of Pediatric Hematology-Oncology, Department of Pediatrics and Child Health, CancerCare Manitoba, University of Manitoba, Winnipeg, CAN; 3 Section of Neurosurgery, Department of Surgery, Winnipeg Children's Hospital, University of Manitoba, Winnipeg, CAN; 4 Section of Neurology, Department of Pediatrics and Child Health, Winnipeg Children's Hospital, University of Manitoba, Winnipeg, CAN; 5 Section of Pediatric Hematology-Oncology, Department of Pediatrics, Montreal Children's Hospital, McGill University Health Center, Montreal, CAN; 6 Section of Neuropathology, Department of Pathology, The Hospital for Sick Children, University of Toronto, Toronto, CAN

**Keywords:** inoperable, molecular biomarker, liquid biopsy, pediatric, low grade gliomas, braf-v600e

## Abstract

Pediatric low-grade gliomas (PLGGs) are the most common central nervous system (CNS) tumors in children. The current standard of care for surgically unresectable and/or progressive cases of PLGGs includes combination chemotherapy. PLGGs are molecularly characterized by alterations in the RAS/RAF/MAPK/ERK pathway in a majority of tumors. PLGGs harboring the BRAF-V600E mutation respond poorly to current chemotherapy strategies. We present a case of a two-year-old female with biopsy-proven low-grade glioma (LGG, pilocytic astrocytoma) involving the hypothalamic/optic chiasm region. At presentation, she had obstructive hydrocephalus, bitemporal hemianopia, central hypothyroidism, and right-sided hemiparesis due to the location/mass effect of the tumor. She was initially treated with chemotherapy (vincristine/carboplatin), but her tumor progressed at six weeks of treatment. She was subsequently started on dabrafenib as her tumor was positive for BRAF-V600E mutation. Dabrafenib monotherapy resulted in dramatic improvement in her clinical symptoms and near-complete resolution of tumor. Our experience and review of the literature suggest that LGGs with BRAF-V600E mutations may benefit from upfront targeted therapy in children. There is an urgent need for prospective clinical trials comparing the efficacy of upfront BRAF inhibitors versus standard chemotherapy in PLGGs with BRAF mutations.

## Introduction

Pediatric low-grade gliomas (PLGGs) are the most common central nervous system (CNS) tumors diagnosed in children, accounting for approximately one-third of cases [1]. While certain PLGGs can be managed effectively with complete surgical resection, others reside in locations that are either not surgically accessible or carry a high operative risk of damaging important neurologic structures [2]. For these tumors, the current standard of care is chemotherapy, typically with a combination of drugs such as vincristine and carboplatin [3]. Radiation therapy is also available for progressive cases, but is typically avoided, when possible, given the associated risks of secondary malignancies and its long-term impact on neurocognitive development [4]. The response of unresectable PLGGs to conventional treatment has been heterogeneous, leading to an increased focus in recent years on studying the molecular pathways involved in tumorigenesis [1]. One pathway that is upregulated in many PLGGs is the mitogen-activated protein kinase/extracellular-signal-regulated kinase (MAPK/ERK) pathway [4,5]. The second most common mutation involving this pathway, which is observed in up to 15-20% of PLGGs, is the BRAF V600E point mutation [4]. Tumors carrying this mutation are often more difficult to treat than those with wild-type (WT) BRAF due to their lack of response to conventional treatment, leading to lower progression-free survival (PFS) and overall survival [4,5]. Novel therapies targeting BRAF, including dabrafenib, have become increasingly used in this population out of necessity for better therapeutic approaches [4,5]. To date, there are very limited features on currently available neuroimaging techniques (computed tomography [CT], magnetic resonance imaging [MRI], and other imaging methods) that can reliably predict a specific molecular diagnosis. This has prompted the emergence of liquid biopsies from biologic samples such as peripheral blood and cerebrospinal fluid (CSF) [2,6-8]. These biofluids may contain material released from cancer cells, including circulating tumor DNA (ctDNA), that can be tested for known PLGG-associated mutations [2,6-8]. This non-invasive method has the potential to play a crucial role in diagnosing PLGGs, providing molecular characteristics of the tumor, monitoring response to treatment, and detecting disease progression [2,6-8]. In this report, we describe a toddler with a large supra-sellar mass that progressed on chemotherapy but demonstrated a significant and sustained reduction on dabrafenib, leading to an almost complete clinical and radiological recovery.

This case report was presented at the 19th International Symposium on Pediatric Neuro-Oncology, ISPNO-2020, Karuizawa, Nagano, Japan.

## Case presentation

**Figure 1:**
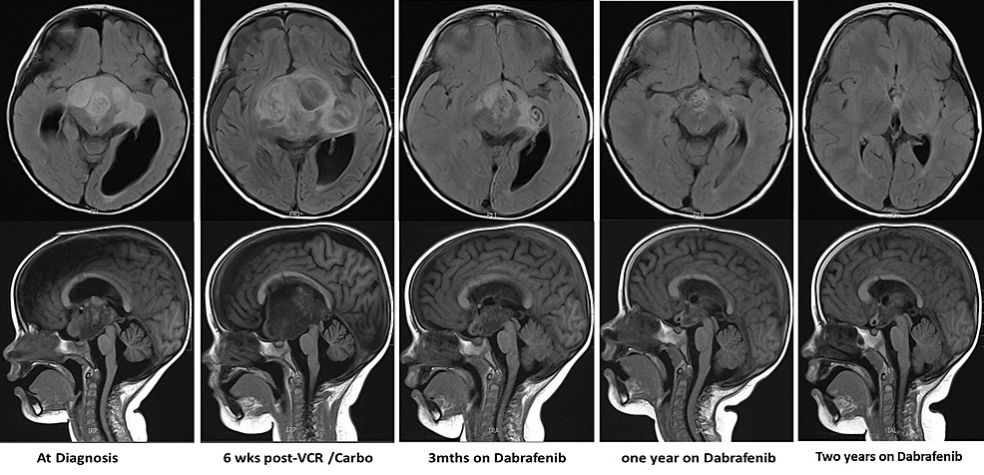
Longitudinal neuro-surveillance with MRI of our patient. Longitudinal surveillance neuroimaging showing T-1 FLAIR MRI images (upper panel: axial; lower panel: sagittal) at various stages of management of the patient. VCR, vincristine; Carbo, carboplatin; FLAIR, fluid-attenuated inversion recovery

**Figure 2:**
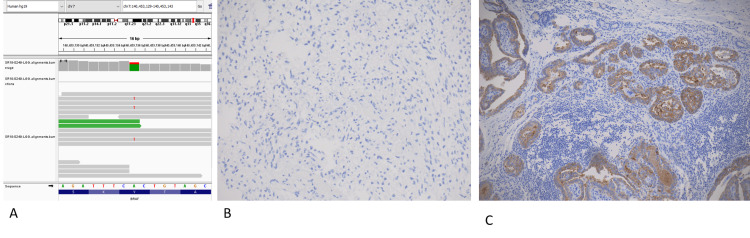
Genomic analysis of patient tumor. A) a genome browser screenshot of targeted RNA sequencing results showing mutation of BRAFV600E. (B) Absence of BRAFV600E immunopositivity by immunohistochemistry in the index case. (C) On-slide positive control (thyroid tumor) shows a strong positivity of BRAFV600E in tumor cells.

A two-year-old female, previously healthy and normally developing, presented with a six-week history of macrocephaly and truncal and peripheral ataxia. An eye examination showed a lack of papilledema but was suspicious for mild peripheral loss of vision. Her past medical history was unremarkable. She did not have diencephalic syndrome at presentation. An urgent brain MRI demonstrated the presence of a large lobulated multicompartmental supra-sellar mass centered within the hypothalamus/optic chiasm (5.4cm x 3.4cm x 5cm) (Figure 1), with extension into the surrounding structures and mass effect on the midbrain and third ventricle causing obstructive hydrocephalus. She initially underwent an endoscopic biopsy and septostomy, along with a right-sided ventriculoperitoneal (VP) shunt to manage her hydrocephalus. The pathology of the lesion confirmed the diagnosis of a low-grade glioma (LGG) that was BRAF-V600E negative on immunohistochemistry but positive on next-generation sequencing (Figure 2). She was started on chemotherapy with vincristine and carboplatin, but unfortunately she had rapid tumor progression with worsening hydrocephalus six weeks into chemotherapy. This progression caused the patient to develop further complications, including progressive right-sided hemiparesis, bitemporal hemianopia, central hypothyroidism, and feeding difficulties requiring a gastrostomy tube and placement of a second VP shunt. After extensive discussions and mutual expert consensus, her chemotherapy was stopped, and she was started on the novel targeted agent dabrafenib (5.25mg/kg/day). By three months of starting dabrafenib, the size of her tumor decreased by more than 70% (2.5cm x 3.5cm x 2.7cm), with continued decline until plateauing after two years of therapy (Figure 1). Prior to dabrafenib, the patient had marked motor and speech impairments but is now able to perform all age-appropriate developmental skills independently. She no longer requires tube feeding and has been growing well. Her bitemporal hemianopia has improved markedly. Her visual acuity is the only symptom that has not shown significant recovery, as she continues to have low but stable visual acuity bilaterally. Our patient is currently five years old, has been on dabrafenib for 30 months, and, to date, she has not experienced any side effects while on targeted therapy. Our plan is to continue on dabrafenib as long as the patient is tolerating the therapy well with no recurrence or progression of the tumor.

Consent was obtained from the parents for sharing of the clinical information and participation in the ORCYD (Oncology Repository for Children and Young Adults) biomarker study. Samples (both blood and CSF) were collected every three months after starting targeted therapy with dabrafenib. Cell-free DNA (cfDNA) from CSF and plasma isolated from blood collected in DNA BCT tubes (Streck, La Vista, NE) were extracted using the QIAamp® Circulating Nucleic Acid kit (Qiagen, Hilden, Germany). Samples were quantified with Qubit™ and the 1X dsDNA high sensitivity (HS) kit (ThermoFisher, Waltham, MA); 1-ul aliquots were loaded on HS DNA chips and run on a Bioanalyzer (Agilent, Santa Clara, CA) to check for cfDNA profile. Samples were pre-amplified with the SsoAdvance™ PreAmp Supermix (Bio-Rad, Hercules, CA) for 10 cycles (annealing temperature = 58°C) following manufacturer's instructions. Diluted pre-amplified samples were mixed with 2X droplet digital polymerase chain reaction (ddPCR) probe mix then used to generate droplets on a QX200 DG (Bio-Rad). Droplets were then submitted to PCR amplification: 95°C for 10 minutes, 94°C for 30 seconds, 56°C for 1 minute (45 cycles), and 98°C for 10 minutes. Droplets were then read on a QX200 Droplet Reader, and data were analyzed using QuantaSoft™ version 1.7 software (Bio-Rad). The primers/probes sequences used for pre-amplification and ddPCR steps are given below:

5’-TCTTCATGAAGACCTCACAGTAA-3’ (BRAF_Fw76pb)

5’-ATGGGACCCACTCCATC-3’ (BRAF_R-long/short)

Wild-type probe: /5HEX/AGATTT+C+A+CTG+T+AGC/3IABkFQ/

Mutant probe: /56-FAM/AGATTT+C+A+CTG+T+AGC/3IABkFQ/

**Figure 3:**
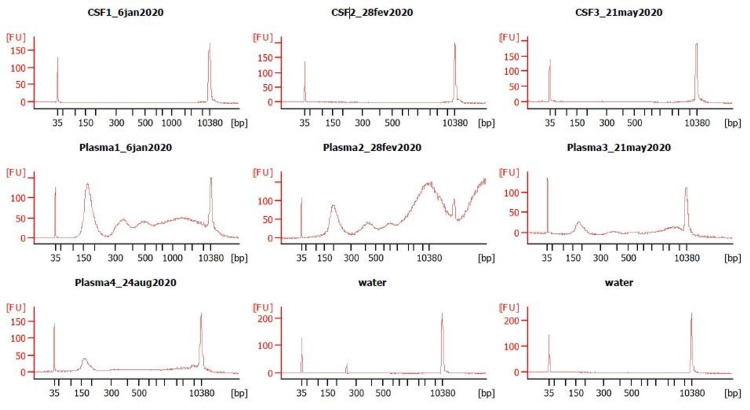
Biomarker analysis by liquid biopsy. Biomarker analysis of BRAF-V600E in CSF (upper panel) and plasma (lower panel): cfDNA separated on the Agilent 2100 Bioanalyzers with an HS DNA chip. Fragment length distribution shows mono-, di-, and tri-nucleosomal peaks, as well as high molecular weight DNA. CSF, cerebrospinal fluid; cfDNA, cell-free DNA; HS, high sensitivity

Three months after starting dabrafenib, the patient was enrolled in the ORCYD biomarker study to test her plasma and CSF for the presence of the BRAF-V600E mutation and to correlate the assay results with the MRI changes observed while on dabrafenib therapy. Samples were collected from the patient (peripheral blood and CSF obtained by lumbar puncture) while sedated for follow-up MRIs over the course of a year. However, the BRAF-V600E mutation was not detected in any of the samples from either plasma or CSF (Figure 3).

## Discussion

LGGs in infants and young children are hugely diverse in their presentation and response to treatment, with the molecular makeup of the tumor playing a significant role in this diversity [9]. Tumors carrying the BRAF-V600E mutation can be found across all pediatric age groups and are more common in midline tumors that originate in the diencephalon and brainstem [1,4]. Not only does this predisposition to developing in midline structures increase the likelihood that BRAF-V600E mutated tumors cannot undergo gross total resection (GTR), but even those that can be resected are more likely to progress compared to those carrying WT BRAF [4]. One study demonstrated that PLGGs with WT BRAF have a five-year PFS of up to 95.9% with GTR and 53.3% with non-GTR, which dropped significantly to 67.8% and 38.8%, respectively, in those with the BRAF-V600E mutation [4]. Those who go on to receive chemotherapy or radiation therapy also have poor outcomes, with tumor control with either modality being achieved in fewer than 30% of patients [4,5]. Given that up to one-fifth of PLGGs may have the BRAF-V600E mutation, it is prudent to develop novel treatment approaches for this unique subset of patients [4].

The dramatic clinical and radiological improvement experienced by our patient while on dabrafenib has been observed in multiple other case reports, including other patients with supra-sellar LGGs (Table 1) [10-14]. All patients achieved a partial response (PR), as defined by a reduction in tumor size on MRI, between 50% and 99%, with most patients having a profound resolution in their neurologic symptoms and a return of their age-appropriate developmental milestones [10-12]. Side effects of BRAF inhibitors are typically minimal, with the most common being mild-to-moderate dermatologic reactions [15]. While very few cases show a complete response to treatment, as defined by complete resolution of the tumor on imaging, these significant reductions in tumor size have demonstrated huge positive impacts on a patient’s quality of life [4,10-12]. The median sustained response time based on short-term studies has been approximately 17 months, which is like the two years of stability experienced by our patient thus far [5]. Most patients who are discontinued on their BRAF inhibitor fail to maintain a sustained PR, with a PFS at one year post-cessation of therapy comparable to those who receive standard chemotherapy [4].

**Table 1:** Literature review. Literature review of patients with midline hypothalamic/supra-sellar low-grade glioma treated with BRAF inhibitors b/l, bilateral

Reference	Age/sex	Tumor location	Genomic findings	Chemotherapy	Targeted therapy	Response	Targeted therapy toxicities
Lassaletta et al. [10]	2 months/male	Hypothalamic/chiasmatic	BRAF-V600E	Vinblastine x 1 week	Dabrafenib	Profound/sustained radiological response	Moderate eczema
						Improvement in vision/weight gain	
						Normalization of neurodevelopment	
Wagner et al. [11]	3 months/male	Hypothalamic/chiasmatic	BRAF: KIAA1549 fusion	Vincristine/carboplatin x 12 weeks	Trametinib	Profound/ sustained radiological response	Nil
						Improvement in vision/weight gain	
				Vinblastine x 12 weeks		Normalization of neurodevelopment	
Bavle et al. [12]	2 months/female	Disseminated: chiasm/b/l optic nerves/thalami/cerebellum	BRAF-V600E	Vincristine/carboplatin x 18 months	Selumetinib x 8 weeks	Partial radiological response	Vesicles/keratosis pilaris/fever
						Improvement in oral intake	
				Vinblastine x 8 weeks	Dabrafenib	Decreased Seizures	
Howden et al. (Current study)	2 years/female	Hypothalamic/chiasmatic	BRAF-V600E	Vincristine/carboplatin x 6 weeks	Dabrafenib	Profound/sustained radiological response	Nil
						Improvement in vision	
						Normalization of neurodevelopment	

The use of targeted therapies in treating pediatric glioma patients carries a lot of promise, but there are still many unanswered questions limiting their application. While short-term side effects have been explored in both pediatric and adult populations, there are no studies to date that have demonstrated the potential adverse effects associated with long-term use. This information is critical given that many patients demonstrate tumor progression once targeted therapy is discontinued, requiring patients to stay on these drugs indefinitely [4]. Additionally, some patients who experience a good response to BRAF inhibitors go on to develop tumor progression despite being on the medication, which was the case for the patient in the study by Bavle et al. [4,12]. This commonly occurs due to accrual of additional mutations in the MAPK/ERK pathway and the development of drug resistance [12,16]. In these circumstances, the optimal treatment approach, including the use of alternative MAPK/ERK pathway inhibitors, remains to be elucidated. Addition of MEK inhibitor at the time of progression on BRAF monotherapy or upfront combination of BRAF and MEK inhibitors is shown to prevent the development of resistance in BRAF-V600E tumors in adults [17].

Exploring the uncertainties associated with BRAF inhibitors with future studies is important given that BRAF-V600E positive PLGGs have poor response rates to chemotherapy. All nonresectable PLGGs currently undergo an initial trial of standard therapies regardless of their mutation status, particularly because of the more robust safety and efficacy data that exist in the literature for current chemotherapeutic regimens [3,18]. However, traditional chemotherapeutic agents employ mechanisms that act in a nondiscriminatory fashion, and preliminary data have shown that BRAF-V600E positive tumors do not behave similarly to PLGGs with other types of BRAF mutations or WT BRAF [4,5]. This highlights that there are certain populations who would potentially benefit from an approach that utilizes BRAF inhibitor therapy upfront instead of trialing current standard therapy first, especially since chemotherapy and radiation therapy often carry significant side effects. Next steps should include the development of clinical trials comparing the use of upfront targeted therapies versus upfront chemotherapy in treating PLGGs carrying the BRAF-V600E mutation, as this could lead to significant improvements in clinical outcomes for this population [18].

Ho et al. describe the distinct radiological characteristics of diencephalic LGGs with BRAF-V600E mutation [13]. The mutant tumors on MRI were multinodular or multiloculated with multifocality at presentation in 25% of the tumors with minimal peritumoral edema, avidly enhancing on T1 sequence post-contrast and T2 abnormalities (suggestive of infiltration) [13]. These findings need to be validated in prospective clinical trials. Liquid biopsies have the potential to detect multiple types of mutations commonly found in PLGGs, including those positive for the BRAF-V600E mutation [2,6-8]. This diagnostic technique has multiple advantages, including being non-invasive, able to summarize the entire genetic makeup of the tumor, and providing quantitative information that can be trended over time and correlated with treatment response [2,6-8]. However, important disadvantages associated with fluid biopsies in PLGGs include the lower level of sensitivity demonstrated by current assays and variability in the sensitivity levels between the various types of biofluids used for this technique [2,8]. With ddPCR, we were not able to detect the presence of tumor-derived genetic material carrying the BRAF-V600E mutation in any of our patient’s seven fluid samples (three CSF and four peripheral blood plasma). This occurred despite collecting samples at multiple timepoints over the span of one year. Of note, we did not collect a sample prior to beginning treatment with dabrafenib, and only obtained our first sample after the patient had already been on targeted therapy for a few months. It is possible that we would have been able to detect the presence of ctDNA using our assay had we sampled the biofluids when the patient was still on chemotherapy, especially since targeted therapies cause such a rapid and effective decline in tumor burden.

PLGGs rarely metastasize outside of the CNS, and the blood-brain barrier can prevent dissemination of tumor-derived genetic material [2]. In our patient’s plasma sample, there was a reasonable number of WT copies detected to get the typical distribution of nucelosomal peaks on the Bioanalyzer (Figure 2). However, despite the sufficient presence of total circulating material quantity, we suspect that there was still a low quantity of ctDNA content, leading to our negative result. One study conducted in adult patients with BRAF-V600E positive malignancies obtained enough ctDNA in peripheral blood to detect the presence of BRAF mutations, but some of the included patients had malignancies that have a greater likelihood of being metastatic and therefore having a higher biofluid ctDNA load [8]. In contrast, a pediatric study conducted on 29 patients with various types of CNS tumors only reported a 25% and 50% sensitivity for detecting ctDNA in plasma and serum, respectively [2]. Unlike the plasma samples, our patient’s CSF only had a few WT copies that could be detected. We therefore hypothesize that the inability to detect mutated tumor DNA for the CSF was likely related to an insufficient quantity of total circulating material. This is reinforced by the fact that we do not see any nucleosomal peaks on the Bioanalyzer traces for CSF samples (Figure 2). For our patient, both CSF and plasma samples were tested with and without preamplification prior to ddPCR.

Quantitation of ctDNA also relies on accurately detecting somatic variants that allow the tumor DNA to be differentiated from other genetic material [6]. PLGGs tend to carry a lower number of mutations compared to other malignancies that have been studied with liquid biopsies, making their detection even more challenging [6]. The type of material being collected also needs to be considered. For example, while certain studies have looked at the quantity of ctDNA, others have looked at the content of cancer mRNA from plasma-derived exosomes (exo-RNA) [19]. It has been demonstrated that biofluid samples with negative DNA results despite tumor progression can have detectable exo-RNA, with the changes in levels correlating with clinical and radiographic findings [19]. One final consideration is the collection method for the CSF tested from our patient. Copy numbers can vary between CSF samples obtained directly from the ventricles versus those obtained with a lumbar puncture like was done in our patient [6,7]. Since she did not have any evidence of spinal disease on MRI, a ventricular sample may have yielded a higher copy number and therefore been more likely to provide a positive result. Ongoing studies assessing the utilization of liquid biopsies in PLGGs with the BRAF-V600E mutation are needed to optimize the process of obtaining and analyzing these biofluid samples to improve their accuracy and utility.

## Conclusions

In summary, BRAF-V600E positive PLGGs in infants and young children are a unique and aggressive subset of tumors that require thoughtful management considerations to improve the clinical outcome of these patients. Many patients with PLGGs have demonstrated a profound and sustained clinical and radiological response to BRAF inhibitors, such as dabrafenib, indicating their potential benefit for both resectable and non-resectable PLGGs. Given the high proportion of patients that fail to respond to the current standard of care therapies, these targeted therapies should be considered in future trials as upfront therapy instead of the current standard of care. In addition, future studies should include further refinement and analysis of liquid biopsies in the diagnosis and management of PLGGs, as these have the potential to provide critical information about the molecular makeup of the tumor without the need for an invasive biopsy and the capability to provide quantitative information over time that can assess treatment response. Ongoing clinical trials with either BRAF inhibitor monotherapy or combination therapy with BRAF and MEK inhibitors upfront (NCT02684058) comparing with chemotherapy will inform the future design and planning of clinical trials with targeted therapies in PLGGs with BRAF-V600E mutations.
